# Mutations F352A and Y528A in human HSP90α reduce fibronectin association and fibrillogenesis in cell-derived matrices.

**DOI:** 10.1007/s12192-023-01362-9

**Published:** 2023-06-23

**Authors:** Abir Chakraborty, Ronald Tonui, Adrienne Lesley Edkins

**Affiliations:** Biomedical Biotechnology Research Unit (BioBRU), Department of Biochemistry and Microbiology, Rhodes University, Makhanda, 6139 South Africa

**Keywords:** Hsp90, Fibronectin, Extracellular matrix, Mutagenesis

## Abstract

HSP90 is a ubiquitously expressed chaperone protein that regulates the maturation of numerous substrate proteins called ‘clients’. The glycoprotein fibronectin (FN) is an important protein of the extracellular matrix (ECM) and a client protein of HSP90. FN and HSP90 interact directly, and the FN ECM is regulated by exogenous HSP90 or HSP90 inhibitors. Here, we extend the analysis of the HSP90 – FN interaction. The importance of the N-terminal 70-kDa fragment of fibronectin (FN70) and FN type I repeat was demonstrated by competition for FN binding between HSP90 and the functional upstream domain (FUD) of the *Streptococcus pyogenes* F1 adhesin protein. Furthermore, His-HSP90α mutations F352A and Y528A (alone and in combination) reduced the association with full-length FN (FN-FL) and FN70 *in vitro*. Unlike wild type His-HSP90α, these HSP90 mutants did not enhance FN matrix assembly in the Hs578T cell line model when added exogenously. Interestingly, the HSP90 E353A mutation, which did not significantly reduce the HSP90 – FN interaction *in vitro*, dramatically blocked FN matrix assembly in Hs578T cell-derived matrices. Taken together, these data extend our understanding of the role of HSP90 in FN fibrillogenesis and suggest that promotion of FN ECM assembly by HSP90 is not solely regulated by the affinity of the direct interaction between HSP90 and FN.

**Supplementary Information:**

The online version contains supplementary material available at 10.1007/s12192-023-01362-9.

## Introduction

Heat shock protein 90 (HSP90) is a member of a highly conserved, widely distributed ATP-dependent chaperone protein family (Taipale et al. 2010; Biebl and Buchner 2019). HSP90 is part of the protein homeostasis network and acts as a key regulator of nascent protein folding, protein refolding, and degradation (Shelton et al. 2017). A large set of intracellular and extracellular protein substrates, known as ‘clients’, interact with HSP90 for optimal activity (Hunter et al. 2014; Wong and Jay 2016). Fibronectin (FN) is a major glycoprotein in the extracellular matrix (ECM) (Pankov and Yamada 2002; Theocharis et al. 2016) and an extracellular client protein of HSP90 (Hunter et al. 2014). The relationship between HSP90 and FN drives pathology associated with diseases like fibrosis and cancer (Chakraborty and Edkins 2021). Fibrosis is characterized by excessive extracellular matrix (ECM) deposition (including FN), hyperproliferative fibroblasts, and inappropriate matrix remodeling (Wynn and Ramalingam 2012; Li et al. 2018; Henderson et al. 2020), which is linked to high levels of extracellular HSP90 that promote ECM stiffness and correlate with disease severity (Sontake et al. 2017; Bellaye et al. 2018). In the context of cancer, high levels of HSP90 in the tumor microenvironment promote matrix remodeling that stiffens stroma, alters FN proteolytic processing and drives cell invasion and metastasis (Kass et al. 2007, Armstrong et al. 2018, Li et al. 2013; Wong and Jay 2016; Baker-Williams et al. 2019). HSP90 inhibition is therefore considered a potential therapeutic strategy to ameliorate the ECM dysregulation characteristic of fibrosis and cancer (Cáceres et al. 2018; Dong et al. 2017; Tomcik et al. 2014; Armstrong et al. 2018).

**Figure 1:**
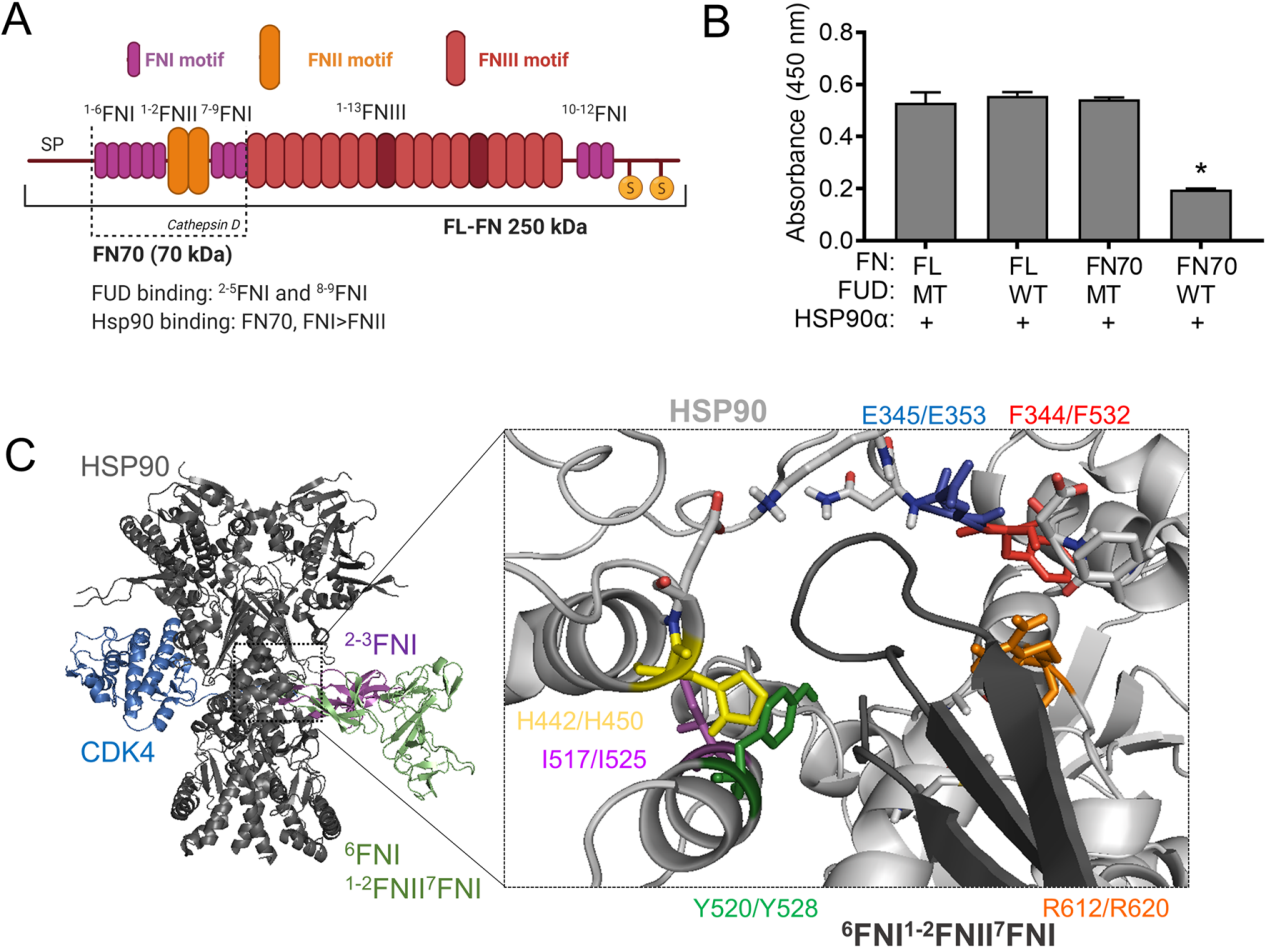
Characterization of the HSP90 interaction with fibronectin. A) Domain structure of full-length fibronectin (FN) and the 70 kDa proteolytic fragment (FN70). Different colors indicate type I (FNI), type II (FNII) and type III (FNIII) FN motifs, respectively. The binding sites of key FN interactors are labelled above, while the sites of proteolytic cleavage of full-length FN are indicated by dotted lines. to the FN70 fragment (^1-6^FNI^1-2^FNII^7-9^FNI) contains key binding sites for FUD and HSP90 B) Interaction of either full-length FN (FN-FL) or FN70 (both at 100 μg/ml; coated on a plate) with HSP90α (2 μM) in the presence of wild type (WT) or mutant (MT) upstream functional domain (FUD) of the F1 adhesin protein from *Streptococcus pyogenes* (2 μM). The binding was detected using anti-HSP90α antibody. Data represent averages (±SD, n=3). Statistical analysis was conducted by two-way ANOVA and Bonferroni post-test comparing the effects of WT and MT FUD for either FN70 or FN_FL, where * p<0.05. C) Docking of the ^6^FNI^1-2^FNII^7^FNI (PDB: 3MQL) and ^2-3^FNI (PDB: 2CG6) modules to HSP90β in the HSP90/CDC37/CDK4 complex (PDB: 5FWK). Overlay of the HSP90β (grey), CDC37 (not shown) and CDK4 (blue) structure with the highest ranked docked models of HSP90β and ^6^FNI^1-2^FNII^7^FNI (green) and ^2-3^FNI (purple). Prediction of putative residues involved in ^6^FNI^1-2^FNII^7^FNI interaction with HSP90β. The loop region from the ^7^FNI motif (dark grey) projects into the interface between the HSP90 dimer at the M domain (light grey). Residues in HSP90β (first number) or topologically conserved in HSP90α (second number) putatively involved in the interaction with ^6^FNI^1-2^FNII^7^FNI were selected for mutagenesis. The colored labels correspond to the color of the residues shown as sticks. Structures were rendered in Pymol (DeLano Scientific).

Structurally, the FN protein is composed of two identical 250 kDa subunits, which are interconnected by a pair of antiparallel disulphide linkages at the C-terminal end (Chauhan et al. 2004; White et al. 2008; White and Muro 2011) (Figure 1). FN is a modular protein made up of repeating units of different motifs which, when combined, form discrete functional domains. These domains support the scaffolding function of FN by allowing spatially distinct interactions with diverse partner proteins through these different motifs and domains. The FN motifs include 12 FN type I repeats (^1-12^FNI), 2 FN type II repeats (^1-2^FNII) and 15 FN type III repeats (^1-15^FNIII) (Figure 1). FN has two distinct forms based on its solubility and tissue-specific distribution. These are soluble plasma FN (pFN), which is synthesised by liver hepatocytes, and less soluble cellular FN (cFN), a heterogeneous group of proteins that arise from a diverse set of alternative splice variants that are synthesised by fibroblasts and smooth muscle cells (To and Midwood 2011). Unlike pFN, dimeric cFN is capable of polymerisation and interacts with cell surface heterodimeric α5β1 and αvβ3 integrin receptors, giving rise to cell-mediated FN assembly, known as fibrillogenesis or matrix assembly. Fibrillogenesis is a complex process that involves the conversion of soluble FN exported from the cell into insoluble matrix-associated FN.

Both isoforms of HSP90 (α/β) have been detected in the extracellular space (Li et al. 2013; Hunter et al. 2014; Mccready et al. 2014; de la Mare et al. 2017), and both interact with FN *in vitro* and in cells. The HSP90 M domain bound to the FN N-terminal domains, showing a preference for interaction with the type I FN motif (FNI) (Hunter et al. 2014; Chakraborty et al. 2020). The N-terminal 30 kDa fragment of FN (FN30) showed the highest affinity for HSP90 (Chakraborty et al. 2020) and is required for FN matrix assembly (Sottile et al. 1991; Maurer et al. 2016). Exogenous HSP90 increased FN matrix assembly (Hunter et al. 2014; Chakraborty et al. 2020), whereas HSP90β inhibition or knockdown resulted in significant FN matrix turnover and decreased matrix stability in Hs578T cells (Hunter et al. 2014; Boel et al. 2020). In prostate cancer explants, HSP90 inhibitor AUY922 also reduced FN secretion (Armstrong et al. 2018), while FN expression was stimulated by HSP90 inhibitor geldanamycin in an HSF1-dependent manner (Dhanani et al. 2017). Here, to understand better how HSP90 promotes FN matrix assembly, we extend the analysis of the HSP90 – FN interaction and identify critical residues involved in HSP90-mediated FN matrix assembly.

## Materials and methods

### Proteins and antibodies

Full-length FN (FL-FN) was from Santa Cruz Biotechnology (SC29011, Dallas, TX, United States). The 70-kDa N-terminal FN fragment (FN70) (F0287) was from Sigma-Aldrich (St. Louis, MO, United States). The details of all antibodies and dilutions used can be found in Supplementary Table 1. Recombinant full-length wild type His-HSP90α, His-HSP90α mutants and wild type and mutant FUD proteins were purified using His affinity column as per our established protocols (Chakraborty et al. 2020). His-HSP90α F352A, E353A, H450A, Y528A, I525A, R620A single point mutants and mutant FUD were generated by site-directed mutagenesis using Q5 mutagenesis kit or generated by Genscript.

### Solid-phase binding protein-protein interaction assay

Solid-phase binding protein-protein interaction assays were performed according to our published protocol with minor modification (Hunter et al. 2014; Chakraborty et al. 2020). Briefly, high-binding plates were coated with 50 μg/mL of either full-length FN (FN-FL) or FN70 fragments, and incubated overnight before blocking with 3% (w/v) bovine serum albumin (BSA) in Buffer A (Hunter et al. 2014). The interacting protein (HSP90 wild type or mutants) was added and incubated overnight at 4 °C. Plates were washed three times with 1% (w/v) BSA in Buffer A and treated with primary antibody in Buffer A. Plates were washed again with 1% (w/v) BSA and incubated with the specific horseradish peroxidase (HRP)-conjugated secondary antibody solution. A 3,3′,5,5′, tetramethylbenzidine TMB substrate solution (0.1 mg/mL TMB in 25.7 mM citric acid, 48.6 mM disodium phosphate, and 0.01% [v/v] hydrogen peroxide) was added to each well and the chemical reaction was ceased by adding 2 M sulphuric acid. Absorbance values were recorded at 450 nm and data were processed using GraphPad Prism software version 4.0.

### Analysis of Cell-Derived Matrices (CDM) by confocal microscopy

The Hs578T breast cancer cell line was maintained in DMEM supplemented with 10% [v/v] FBS, 2 mM GlutaMAX™, 100 U/mL PSA, and 2 mM insulin (Novo Nordisk A/S, Bagsværd, Denmark) at 37 °C in a humidified chamber with 9% CO_2_. Protocols for ECM production and harvest from Hs578T breast carcinoma cells were adapted from published protocols (Fernandez-Garcia et al. 2014; Chakraborty et al. 2020). Briefly, ethanol-sterilized coverslips in a 24-well plate were incubated with 0.2% [w/v] sterile gelatin for 1 h at 37 °C. Gelatin crosslinking was performed using 1% [v/v] sterile glutaraldehyde in PBS for 30 min at room temperature. Wells were washed with PBS and the crosslinker were quenched with 1 M sterile ethanolamine. Wells were again washed three times with PBS before seeding cells onto the prepared coverslips in 24-well plates (at 80% confluency). After reaching confluency, the medium was replaced with medium containing 50 μg/ml ascorbic acid. This treatment was repeated every second day. After 6 days of culture, cells were treated for 48 h with 200 ng/ml recombinant endotoxin-free HSP90 or FUD proteins. Next, the cells were washed with PBS and treated with 50 mM EDTA for 10 min at 37 °C. Cells were washed twice with PBS and incubated with the extraction buffer (20 mM NH_4_OH and 0.5% [v/v] Triton-X in PBS) preheated to 37 °C until cell lysis. Next, without replacing the extraction buffer, PBS was added to each of the wells, and the plate was incubated at 4°C overnight to improve the stability of the newly extracted matrices. Wells were treated with 10 μg/mL of DNase I (Roche, Basel, Switzerland) for 30 min at 37 °C, washed three times with PBS and cells fixed with 3.7% [w/v] paraformaldehyde solution.

Fixed cell matrices were blocked with 1% [w/v] BSA/PBS for 40 min followed by incubation with primary antibodies in 1% [w/v] BSA/TBS overnight at 4 °C. The following day, coverslips were washed twice with 1% [w/v] BSA/TBS-T (TBS containing 0.1% [w/v] Tween-20) followed by 1 h incubation with species-specific fluorescently tagged secondary antibodies (see Supplementary table 1). Nuclei were stained with Hoechst 33342 dye (Invitrogen) (1 μg/ml in distilled water). Images were captured using the Zeiss LSM780 Meta laser scanning confocal microscope and analyzed using Zen Blue Software. The intensity of the FN matrix signal was quantified from confocal images by determining the average integrated density of the signal in ImageJ from multiple independent frames of the same size.

### Differential scanning fluorimetry assay

The differential scanning fluorimetry assay was adapted from the published protocol using the BioRad CFX Connect thermal cycler system (Huynh and Partch 2015). Briefly, a final 5X concentration of SYPRO Orange dye was mixed with 5 μM of protein (HSP90α wild type or mutants) in a total 50 μl reaction volume in standard 25 mM sodium phosphate buffer. A stepwise temperature increment (0.7 ^0^C) from 25 °C to 95 °C with 1 min hold times and an initial 2 min hold time was set and fluorescence signal was recorded using the VIC filter set. The melting temperature (T_m_) of unfolding was calculated from raw fluorescence values using GraphPad prism 4.

### Statistical analysis and reproducibility

Data are representative of at least 3 independent biological experiments unless otherwise stated. Statistical analysis was performed by either one-way ANOVA and Tukey’s multiple comparison test or two-way ANOVA and Bonferroni post-test in GraphPad Prism 4.0, where * p<0.05 considered significant.

## Results and Discussion

### HSP90 and FUD compete for binding to FN

The functional upstream domain (FUD) protein is a 49 amino acid domain in the F1 adhesin protein of *Streptococcus pyogenes.* The FUD binds to the ^2-5^FNI and ^8-9^FNI repeat regions in the FN70 fragment by β-strand addition. The FN70 domain is required for FN fibrillogenesis, and FUD binding prevents FN matrix assembly (Maurer et al. 2010). Similar to FUD, HSP90 also bound the FN70 fragment and preferentially recognised the FNI motif *in vitro* (Chakraborty et al. 2020) (Figure 1A). HSP90 also regulates FN fibrillogenesis, but in contrast to FUD which blocks FN fibrillogenesis, exogenous HSP90 promoted FN matrix assembly (Hunter et al. 2014; Chakraborty et al. 2020). To analyse the effect of FUD on the HSP90 interaction with FN, we purified recombinant wild type FUD (WT FUD) (which inhibits FN assembly) and a mutant FUD variant (MT FUD) (that does not prevent FN matrix assembly). MT FUD did not alter the interaction of His-HSP90α with either full-length FN or FN70 (Figure 1B). The interaction of FN70 and HSP90α was reduced in the presence of WT FUD, but there was no effect on the interaction of HSP90 with FN-FL (Figure 1B). This is most likely because FUD has a tenfold stronger binding affinity for FN70 (K_D_ 5.2 nM) than FN-FL (K_D_ 59 nM) (Maurer et al. 2010). These data reinforce the importance of the FNI repeat for HSP90 binding and indicate that the HSP90 binding site overlaps with the ^2-5^FNI and ^8-9^FNI repeats in the FN70 fragment bound by FUD.

### Residues F352 and Y528 in the HSP90 M domain are involved in the FN interaction.

We next attempted to identify the interface residues in the HSP90M domain involved in the FN interaction. To do this, we first conducted *in silico* docking with the ClusPro 2.0 server (Kozakov et al. 2017) using available HSP90 and FN structures and compared the residues predicted to be involved in the interaction with those required for client interactions in published studies (Figure 1C). HSP90 preferentially bound the FNI repeat motif over the FNII (Chakraborty et al. 2020) and so we docked FN structures comprising ^6^FNI^1-2^FNII^7^FNI (PDB 3MQL) and ^2-3^FNI (2CG6) (Rudiño-Piñera et al. 2007; Erat et al. 2010) to the cryo-EM structure of HSP90β in complex with the client protein CDK4 and CDC37 (PDB 5FWK) (Figure 1C). In this structure, putative CDK4 residues involved in contacting HSP90β included Glu345 (E345), Phe344 (F344), Ile517 (I517), Tyr520 (Y520), Arg612 (R612), Met602 (M602), and Leu611 (L611) (Verba et al. 2016).

The majority (~80%) of models returned showed the docking of the ^6^FNI^1-2^FNII^7^FNI and ^2-3^FNI structures to the middle domain of HSP90, within a region similar to that occupied by CDK4 (Verba et al. 2016). The predicted binding to the M domain was consistent with our *in vitro* interaction analysis (Chakraborty et al. 2020)**.** For both the ^6^FNI^1-2^FNII^7^FNI and ^2-3^FNI models, the FN region predicted to be involved in HSP90 interaction was a loop region within a β sheet of an FNI type motif. This loop projected into a groove in the M domain at the interface of the HSP90 dimers, consistent with CDK4 binding (Figure 1C). In the ^2-3^FNI model, loops from both the ^2^FNI and ^3^FNI motifs were predicted to dock to the M domain of HSP90. In the ^6^FNI^1-2^FNII^7^FNI, the predominant docking was predicted to a loop within the ^7^FNI.

**Figure 2:**
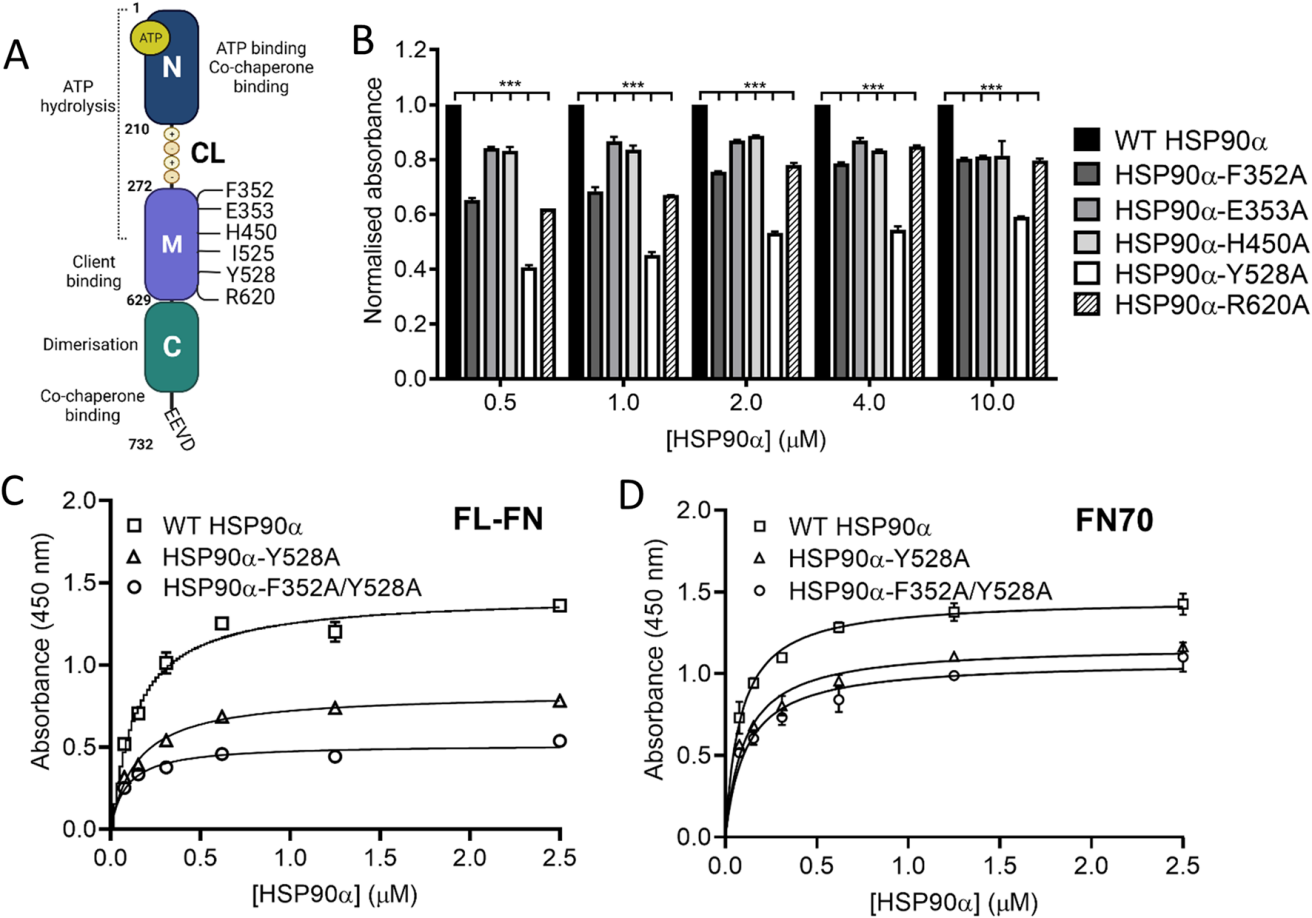
Mutations in the HSP90 M domain alter interaction with fibronectin. A) Schematic diagram of full-length HSP90. N, M and C indicates N-terminal, Middle and C-terminal domains, respectively. Region of ATP binding and hydrolysis is indicated with the dotted line whereas numbers in the middle domain refer to key mutations used in this study. B) & C) Solid-phase binding assay for the interaction of full-length FN (25 μg/ml; coated on plate) with wild type (WT) or mutant His-HSP90α. (D) Interaction of FN70 (25 μg/ml: coated on plate) with varying concentrations of wild type (WT) or mutant HSP90α WT. Binding was detected using an anti-HSP90α antibody. Statistical analysis was conducted by two-way ANOVA and Bonferroni post-test comparing the wild type HSP90α to each of the mutant proteins, where * p<0.05.

Using the ^6^FNI^1-2^FNII^7^FNI models, we subsequently identified HSP90β residues (and corresponding residues in HSP90α) within 4 Å of the putative interacting FNI loop regions (Figure 1C). Residues F341 (F349), D342 (D350), F344 (F352), E345 (E353), N346 (N354), K347 (K355), H442 (H450), E443 (E451) Y520 (Y528), M606 (M614), A610 (A618), R612 (R620), and M620 (M628) in HSP90β (or HSP90α in brackets) were predicted to be in proximity to the loop region (Figure 1C). Residues F352, E353, I525, Y528, and R620 had previously been predicted to be involved in HSP90-client protein interactions from other studies (Verba et al. 2016). We therefore introduced alanine substitutions into the 6 topologically equivalent residues in HSP90α, namely F352A, E353A, H450A, Y528A, and R620A (Figure 2A). In addition, the I525A (I517A in HSP90β) mutation was included because it was predicted to be a critical residue in the binding interface between the client protein CDK4 and HSP90 (Verba et al. 2016).

The HSP90α-F352A, HSP90α-E353A, HSP90α-H450A, HSP90α-I525A, HSP90α-R620A, and HSP90α-F352A/I525A proteins were purified from *E. coli* with yields between 24 mg/L and 30 mg/L, while purified HSP90α-Y528A and HSP90α-F352A/Y528A proteins had yields between 12 mg/L and 15 mg/L (data not shown).We performed a thermal stability shift assay on the His-HSP90α mutants to confirm if the mutations had a significant impact on the His-HSP90α stability. Except for the His-HSP90α-I525A mutant, all the mutant HSP90α proteins had T_m_ values similar to the wild type His-HSP90α and consistent with published values (Chadli et al. 1999). The His-HSP90α-I525A mutant was substantially less stable than the wild type His-HSP90α protein, as indicated by its reduced T_m_ and hence was excluded from any further analysis (Table 1).

**Table 1:** Differential scanning fluorimetry of Hsp90 variants

HSP90 variants	T_m_ ± SD (n = 3)
His-HSP90α Wild type	50.4 ± 0.00
His-HSP90α-F352A	50.4 ± 0.00
His-HSP90α-E353A	48.3 ± 0.00
His-HSP90α-H450A	49.0 ± 0.00
His-HSP90α-I525A	39.0 ± 1.60
His-HSP90α-Y528A	51.1 ± 0.01
His-HSP90α-R620A	49.7 ± 0.01
His-HSP90α-F352A/Y528A	49.2 ± 0.50

Of the 5 mutations, the His-HSP90α-Y528A had the biggest impact on the HSP90 – FN interaction, reducing the interaction by ~50% of that of the wild type His-HSP90α (Figure 2B). The His-HSP90α-F352A mutant showed ~40% reduction in binding compared to wild type His-HSP90α at the lower concentrations. All the other His-HSP90α mutations (E353A, H450A, Y528A and R620A) resulted in ~25% reduction in binding to FN compared to wild type His-HSP90α (Figure 2B). We subsequently generated a double mutation to combine the single mutations which resulted in the greatest reduction in FN-His-HSP90α interaction (namely F352A and Y528A). The His-HSP90α-F352A/Y528A showed significantly reduced binding to FN-FL, below that observed for the His-HSP90α-Y528A single mutant (Figure 2C).The His-HSP90α-Y528A and His-HSP90α-F352A/Y528A also showed significantly reduced interaction with FN70 compared to the wild type His-HSP90α. Similar to FN-FL, the double mutation of F352A and Y528A reduced the His-HSP90α interaction more than the His-HSP90α-Y528A single mutant (Figure 2D).

### HSP90 M domain residues F352, E353 and Y528 are involved in HSP90-mediated FN fibrillogenesis.

**Figure 3:**
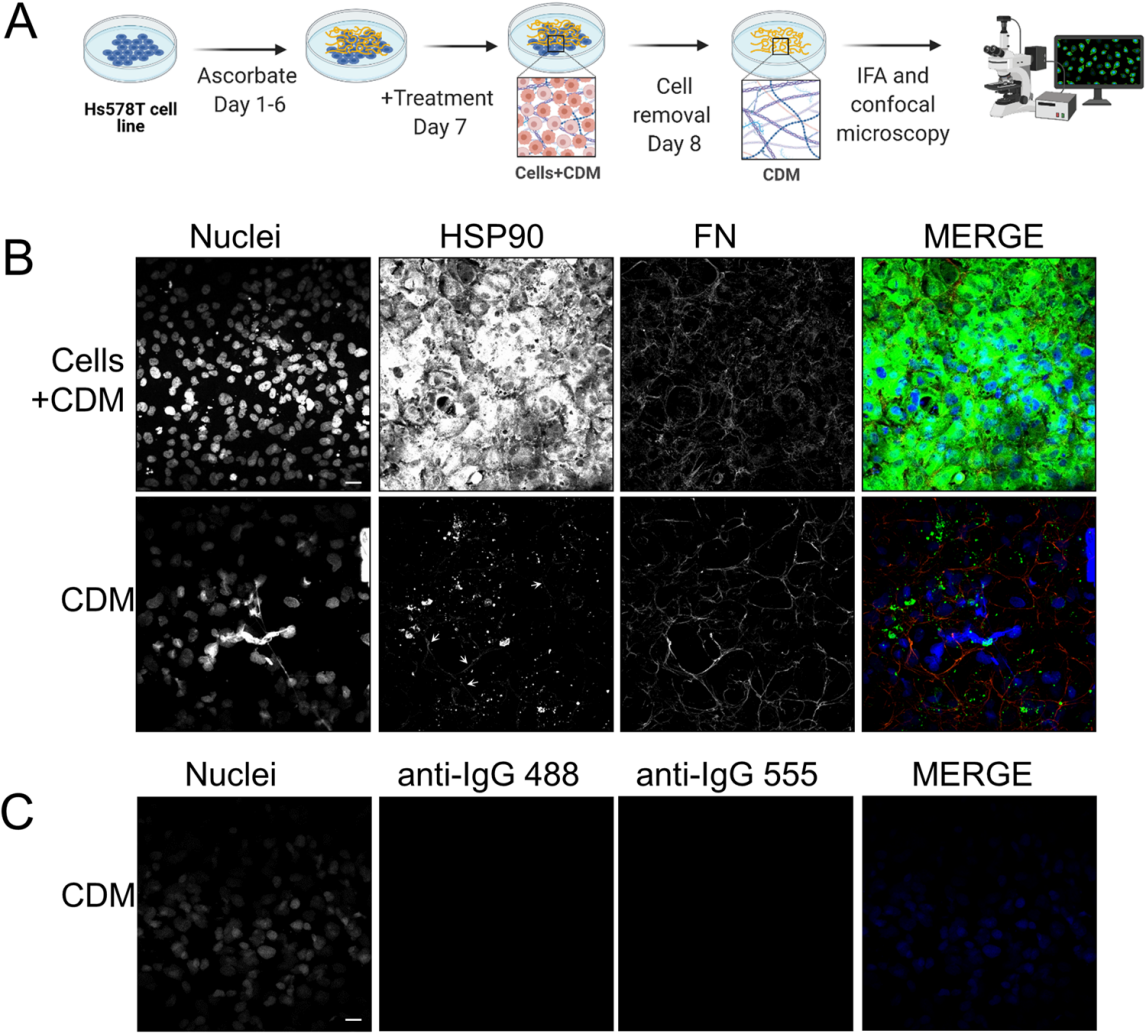
Analysis of Hs578T cell-derived matrices *in vitro.* (A) Schematic diagram of the process to generate cell-derived matrices (CDM). Hs578T cells, which endogenously produce and assemble FN matrix, were cultured for 6 days in the presence of ascorbate to stimulate matrix production. On day 7, cells were treated with recombinant exogenous endotoxin-free proteins overnight. The following day, the matrices were treated to remove cell-associated proteins and partial removal of DNA. The remaining cell-derived matrices (CDM), which represent insoluble matrix-associated proteins, were analysed by confocal microscopy after immunostaining for FN. (B) Comparison of typical staining patterns for FN (red) and endogenous HSP90α (a marker of the cytoplasm; green) in Hs578T samples before removal of cells (Cells + CDM) and in cell-derived matrices (CDM) after cell removal. The specificity of the immunostaining in CDM was confirmed by controls in which only the fluorescently labelled secondary antibodies were used in immunostaining. Scale bars indicate 10 μm.

The N-terminal fragments of FN that bind HSP90 are required for FN matrix fibrillogenesis (Schwarzbauer and DeSimone 2011) and exogenous HSP90 promotes FN fibril formation (Hunter et al. 2014; Chakraborty et al. 2020). Therefore, we asked whether the interaction between extracellular HSP90α and FN was required for HSP90-mediated FN matrix assembly. We analyzed the effect of exogenous, recombinant, endotoxin-free HSP90α on the production of insoluble FN matrices *in vitro*. We made use of the Hs578T breast cancer cell line, which endogenously produces and assembles detectable levels of FN matrix *in vitro* to assess the transition of soluble cell-associated FN into an insoluble cell-derived matrix containing FN fibrils (Hunter et al. 2014). Hs587T cells were treated to promote FN matrix assembly for 6 days and thereafter treated for 48 h with exogenous recombinant endotoxin-free HSP90α or controls. The following day, the cells were removed and the remaining CDM analyzed for FN by immunofluorescence staining and confocal microscopy (Figure 3A). We confirmed that our protocol was working by comparing the staining of FN (which is detected in the cytoplasm and as extracellular fibrils) and endogenous HSP90α (which is predominantly detected in the cytoplasm) in both untreated Hs578T cell cultures (Cells+CDM) and the insoluble Hs578T-derived matrix (CDM) after removal of cell-associated proteins (Figure 3B). In the untreated cell cultures, the HSP90α signal was detected throughout the cytoplasm. The FN staining pattern showed diffuse staining that was a mixture of fibrils and cytoplasmic staining. After removal of cells, the FN fibrils in the stained CDM (representing insoluble matrix-associated FN) were more prominent. The HSP90α staining in the CDM showed bright puncta, which likely represent residual cell-associated HSP90α. In addition, HSP90α was detected in fibrils which potentially represents matrix-associated HSP90α in FN fibrils (Figure 3B, white arrows). The loss of a substantial cytoplasmic signal for HSP90α indicated that the decellularization protocol was successful. While some residual DNA staining was observed, there was a change in nuclear morphology consistent with cell lysis. The staining detected in the CDM was shown to be specific for FN and HSP90α as the controls of secondary antibodies alone did not show any signal (Figure 3C).

**Figure 4:**
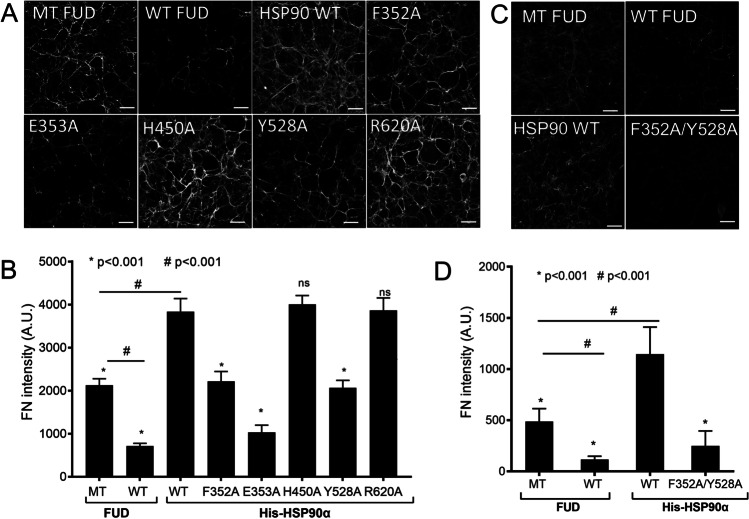
HSP90α mutants Y528, F352 and E353 do not promote FN matrix assembly. A) & C) Immunofluorescence detection and confocal microscopy analysis of FN fibrils in Hs578T cell-derived matrices (CDMs) after treatment with either the upstream functional domain (FUD) of the F1 adhesin protein from *Streptococcus pyogenes* or His-HSP90α protein variants. MT FUD: mutant FUD mutant, WT FUD: wild type FUD sequence. Images shown are representative of 3 independent images showing similar intensity and morphology. B) & D) Quantitation of the average (±SD, n=3) FN fibril signal intensity using ImageJ. Statistical analysis was conducted in Graph Pad prism 4.0 by one-way ANOVA and Tukey’s multiple comparison test, where * p<0.001 when comparing WT His-HSP90α with all other treatments and # p<0.001 when comparing MT FUD with all other treatments.

Next, we tested the effect of recombinant, exogenous, wild type and mutant HSP90α proteins on the formation of FN fibrils using the optimized Hs578T CDM assay (Figure 4). We had previously identified endogenous HSP90α in FN containing fibrils (Figure 3B). Therefore, we predicted that the exogenous HSP90 variants would either synergize with or inhibit the activity of existing endogenous HSP90α. The wild type FUD protein binds FN and blocks FN fibrillogenesis and therefore was used as a control for inhibition of fibrillogenesis, leading to reduced FN fibrils in the CDM (Maurer et al. 2010). We used the mutant FUD protein, which does not inhibit FN fibrillogenesis, as a control for the presence of additional protein on FN fibrillogenesis.

The Hs578T cells treated with the control FUD mutant contained FN fibrils indicating the formation of FN CDM in these cells (Figure 4A and B). This provided the baseline for FN CDM levels in cells treated with exogenous protein that does not alter FN fibril formation. Wild type FUD protein resulted in a significant reduction in FN fibril and CDM compared to the mutant FUD control (Figure 4A and B). Wild type exogenous His-HSP90α treatment promoted FN fibrillogenesis as indicated by the significantly increased fibril formation and intensity compared to the FUD mutant control (Figure 4A and B). This is consistent with the increased FN fibrils previously identified in Hs578T cells treated with exogenous HSP90β (Hunter et al. 2014). The His-HSP90α-F352A and His-HSP90α-Y528A mutants showed significantly lower levels of FN CDM compared to the wild type His-HSP90α and resembled the FUD mutant control. The His-HSP90α-H450A and His-HSP90α-R620A mutants significantly promoted FN fibril formation compared to the FUD mutant and resembled wild type HSP90α. The His-HSP90α-E353A mutant significantly inhibited FN fibril formation similar to WT FUD (Figure 4A and B). In addition, we tested the effect of the His-HSP90α-F352A/Y528A double mutant on FN matrix assembly (Figure 4C and D). The His-HSP90α-F352A/Y528A mutant showed a significant reduction in FN matrix assembly compared to wild type His-HSP90α, and mutant FUD treated cells (Figure 4C and D). However, even though the His-HSP90α-F352A/Y528A double mutant showed reduced FN association compared to the His-HSP90α-Y528A single mutant (Figure 2C and D), there was no significant difference in the FN CDMs between these samples (data not shown).

The changes in FN fibril intensity did not directly correlate with the interaction of His-HSP90α mutants with FN (Figure 2B). The His-HSP90α-F352A mutant which showed ~20% reduction in FN interaction had the same effect on the FN ECM as His-HSP90α-Y528A mutation which showed ~50% reduction in interaction with FN. The His-HSP90α-F352A and the His-HSP90α-R620A both showed ~20% reduction in interaction with FN, but the His-HSP90α-F352A mutant failed to promote FN ECM assembly, while the His-HSP90α-R620A mutant resembled the wild type His-HSP90α. Additionally, the His-HSP90α-E353A mutant, which did not show substantially altered interaction with FN (Figure 2B), significantly inhibited FN fibril formation and resembled the wild type FUD treated CDMs (Figure 4A and B). In addition to influencing client and co-chaperone interactions, mutations in the M domain also alter the ATPase activity of HSP90 (Hawle et al. 2006; Xu et al. 2019), which could be verified for these mutations by *in vitro* ATPase assays. However, while HSP90 ATPase activity is critical for intracellular client protein maturation, the requirement for ATPase activity in the extracellular environment has not been clarified, with data suggesting that ATPase activity is both involved and is expendable (Li et al. 2013).

HSP90 undergoes post-translational modifications (PTM) including phosphorylation, oxidation, acetylation, S-nitrosylation, methylation, SUMOylation and ubiquitination (Scroggins and Neckers 2007; Mollapour and Neckers 2012; Backe et al. 2020). PTMs play a pivotal role in HSP90 chaperone cycle regulation and tailoring the client binding and activity, a phenomenon termed the “chaperone code” (Backe et al. 2020). These PTM in turn can culminate in changes in cancer migration, invasion and tumorigenicity (Xiaofeng et al. 2009; Dagar et al. 2019). Phosphorylation is the most widely studied HSP90 PTM (Mollapour and Neckers 2012), affecting chaperone activity directly or allosterically via co-chaperone and client dynamics. For example, tyrosine phosphorylation on HSP90 greatly increased eNOS interaction (Brouet et al. 2001), whereas GA-treated HSP90 had reduced tyrosine phosphorylation which decreased HSP90-P2X7 receptor association and increased p2X7 agonist sensitivity (Adinolfi et al. 2003). In our study, the Y528 residue was critical for HSP90 interaction with FN, where the exogenous application of HSP90 mutant Y528A showed reduced FN interaction and reduced FN fibrillogenesis. Although phosphorylation of this residue has yet to be reported experimentally, it is possible that Y528 phosphorylation in the extracellular space is involved in mediating the HSP90-FN interaction. Phosphorylation of extracellular proteins in the ECM by secreted kinase c-Src has been demonstrated recently (Sánchez-Pozo et al. 2018) and c-Src is known to phosphorylate Y300 on HSP90 (Duval et al. 2007). The potential contribution of phosphorylation of Y528 to FN dynamics could be studied in future using a phosphomimetic mutant (Y528E), producing HSP90 in mammalian expression systems and studying effects of kinase inhibition or RNAi-mediated depletion.

Collectively, these data indicate that exogenous HSP90α promotes FN fibril formation, and that the interaction between FN and HSP90α is consistent with a role in regulating fibrillogenesis. However, the strength of the interaction between FN and His-HSP90α is not the sole determinant of enhanced FN fibrillogenesis due to exogenous His-HSP90α. This suggests that the promotion of FN matrix assembly is governed by other features of extracellular His-HSP90α beyond the interaction with FN. Interestingly, the pro-fibril forming effect of HSP90 was opposite to the effect induced by FUD, wherein wild type FUD reduced matrix assembly. HSP90 and FUD compete for binding to the same FN70 domain, which suggests overlapping binding sites but different outcomes of that interaction. This implies that different FN interacting partners may either promote or inhibit matrix assembly through interaction with a common FN motif or fragment. While FUD may bind FN70 in an inhibitory mode that blocks matrix assembly, HSP90 binding to the same FN70 fragment activates matrix assembly (Tomasini-Johansson et al. 2001). It would be interesting to determine in future if the FUD protein could prevent HSP90-mediated increases in the FN matrix.

## Conclusion

Our data provide novel insight into the understanding of HSP90 client protein interactions and are to the best of our knowledge, the first study of this nature to focus on an extracellular client protein. Our analysis predicts that FN may share a similar binding interface with cyclin-dependent kinase 4 (CDK4) (Verba et al. 2016). The functional upstream domain (FUD) of the *Streptococcus pyogenes* F1 adhesin protein binds via β-strand addition to the ^2-5^FNI and ^8-9^FNI repeats in the N-terminal FN70 domain and blocks matrix assembly (Maurer et al. 2010). Wild type FUD and HSP90 compete for binding to FN70, reinforcing the importance of the FNI motif for the HSP90 – FN. The pro-fibril forming effect of HSP90 was opposite to the effect induced by FUD, wherein wild type FUD reduced matrix assembly. The pro-fibrillogenic role of HSP90α is consistent with recognition of the N-terminal FN domains, as these domains are required for FN ECM assembly. Notably, residues Y528, F352 and E353 are important for stimulation of FN ECM production by exogenous His-HSP90α. Furthermore, the ability of eHSP90α to promote FN fibrillogenesis is an important mechanistic insight into the role played by HSP90 in diseases like fibrosis and cancer, where FN fibrillogenesis is altered and drives pathology. Our data suggest that because the interaction between HSP90 and FN is important in promoting fibrillogenesis, in addition to pharmacological inhibition of HSP90, specific disruption of the HSP90-FN interaction may be a strategy to reverse ECM dysregulation. The importance of these specific residues in HSP90 for FN assembly may allow selective targeting of this region of HSP90 as a putative therapeutic strategy for disorders like invasive cancers and fibrosis in the future.

### Supplementary Information

Below is the link to the electronic supplementary material.

Supplementary file1 (DOCX 14.2 KB)
