# Supplementary material for: Mutations F352A and Y528A in human HSP90α reduce fibronectin association and fibrillogenesis in cell-derived matrices
Source: Cell Stress Chaperones. 2023 Jun 23;28(6):697–707. doi: 10.1007/s12192-023-01362-9 (PMC10746679; doi:10.1007/s12192-023-01362-9)
Supplement: Supplementary file 1 — Supplementary file1 (DOCX 14.2 KB) [file 12192_2023_1362_MOESM1_ESM.docx]

Supplementary Table 1: Key resources table

| Name | Description | Source |
| --- | --- | --- |
| Anti-His antibody | Mouse monoclonal anti-His antibody | SC51946 (1:1000 dilution) |
| Anti-FN antibody | Rabbit polyclonal antibody | Sigma-Aldrich F3648 (1:5000 dilution) |
| Anti-HSP90A antibody | Rat monoclonal anti-HAP90α antibody against clone 9D | ADI-SPA-840 (1:10000 dilution) |
| Donkey anti-mouse IgG H&L Alexa fluor 488 | Fluorophore-conjugated secondary antibody | Ab150105 (1 in 500 dilution) |
| Donkey anti-rabbit IgG H&L Alexa fluor 555 | Fluorophore-conjugated secondary antibody | Ab150074 (1 in 500 dilution) |
| Donkey anti-rat IgG H&L Alexa fluor 488 | Fluorophore-conjugated secondary antibody | Ab150153 (1 in 500 dilution) |
| HRP-conjugated anti-mouse secondary antibody | HRP-conjugated secondary antibody | A16011 (1:5000 dilution) |
| pET-16b-HSP90α-WT | Plasmid for production of His-HSP90α-WT in *E. coli* | This study |
| pET-16b-HSP90α-F352A | Plasmid for production of His-HSP90α-F352A in *E. coli* | This study |
| pET-16b-HSP90α-E353A | Plasmid for production of His-HSP90α-E353A in *E. coli* | This study |
| pET16b-HSP90α-H450A | Plasmid for production of His-HSP90α-H450A in *E. coli* | This study |
| pET-16b-HSP90α-Y528A | Plasmid for production of His-HSP90α-Y528A in *E. coli* | This study |
| pET-16b-HSP90α-R620A | Plasmid for production of His-HSP90α-R620A in *E. coli* | This study |
| pET-16b HSP90α-F352A/Y528A | Plasmid for production of His-HSP90α- F352A/Y528A in *E. coli* | This study |
| pCA528-FUD-WT | Plasmid for production of His-Sumo-FUD-WT in *E. coli* | This study |
| pCA528-FUD-MT | Plasmid for production of His-Sumo-FUD MT in *E. coli* | This study |
| FN-FL | Human full-length fibronectin protein | SC29011 (Santa Cruz  Biotechnology) |
| FN70 | 70 kDa N-terminal FN fragment produced by cathepsin D treatment of full-length FN (1-6FNI1-2FNII7-9FNI; referred to as FN70) | F0287 (Sigma) |
